# The Practice of Prevention

**Published:** 1919-10-25

**Authors:** 


### The Hospital

The Workers' Newspaper of Administrative Medicine and Institutional Life, Administration, National Insurance and Health.

No. 1742, Vol. LXVII. SATURDAY, OCTOBER 25, 1919. PRICE TWOPENCE

### THE PRACTICE OF PREVENTION.

Although we must accept the recently published memorandum on Preventive Medicine prepared by Sir George Newman at the request of the Ministry of Health as the first general statement of policy and objects by the new department, yet it will be well if the members of that immense army of workers, upon whose efforts success or failure in these schemes will depend, remember that however greatly our knowledge of hygienic matters has advanced in recent times, there is an immense amount of preliminary work to be done before this knowledge can be in any sense efficiently applied. The aims of the Ministry appear to fall under three main headings. There is the individual himself, his physique, to be improved so that collectively the working capacity and resistance of the community to disease may be increased. Next comes direct attack upon the multitude of causes and conditions favourable to disease; and last, the attempt to prolong the life of the average individual. A vast objective, but one requiring preliminaries of yet greater magnitude.

In years now past, the State concerned itself mainly with acutely infectious disease, the control of nuisances, and a very necessary supervision of certain more or less dangerous trades, but to carry out such a comprehensive policy as that now stated has never before been attempted. Yet, in spite of this neglect, we read that the nation has become steadily more healthy and the length of human life in this country has increased, a tendency which can be traced, in part at least, to the development and labours of our voluntarily supported hospitals with their output of trained doctors and nurses, and to a gradual process of enlightenment among the general public. Well may we congratulate ourselves upon this decided improvement on the nation's health during the past few decades, but at the same time it must be realised that in a very few years not only will the population be vastly more crowded than it is to-day, but also that the stress and strain upon the individual threatens correspondingly to increase. Unless the intensity of this process be counteracted by progress along such lines as those now indicated by Sir George Newman, the greatest wealth of the nation, its people's health, must sooner or later inevitably decline.

If further evidence be needed, let us note the fact that the number of recruits placed, during the late war, in the lowest categories of ill-health amounted to practically one million men. Also that there is a steadily falling birth-rate, and that each year approximately half a million persons die before reaching the age of fifty years. In proof of present inadequacy we read that in one year there were about one million cases of measles in the country, in the same year tuberculosis claimed 92,000 fresh victims and 6,500 newly-born children developed ophthalmia. Half the school children in the country are still in need of dental treatment, half a million are in urgent need of it and as many more are so defective in eyesight as to be unable to take reasonable advantage of their lessons.

Sir George Newman justifiably points out that although we have access to an immense amount of medical knowledge, experience old and new, and a satisfactory strength of desire to apply it to these matters, yet there is a lack of correlation of the knowledge, a lack of understanding of the precise problems to be solved and of the exact ways and means by which they may be faced, and this applies very markedly to the practitioners of the country. It is not the skilled consultant who is necessarily in the forefront in this fight with ill-health, but the more humble but invaluable general practitioner. To him and his successors it is urgent that they should fully grasp the principles of preventive medicine, the practice of this art, and so automatically provide that co-operation without which the schemes of the Ministry must fail. The country needs more doctors and, in one sense, "better doctors." The newest plans of our medical schools are fortunately attuned to this requirement, and we trust that the new era will produce a profession well able to answer the most exacting demands from a growing department.

One great point Sir George Newman would impress, and it is applicable to almost every phase of life. There is still "a vast mass of disability and incompetency from widespread maladies usually regarded as trivial and negligible." They are mostly unmentioned in our official records, often unheeded by the profession even when encountered. Yet all exert their influence, and that a marked one, in producing numerous unsatisfactory health statistics to which this memorandum draws attention. These simple conditions hold among their number many, in fact most, of the very earliest signs of disease, and one of the first and most vital movements in preventive medicine must be that the public and profession alike should realise their meaning.

So might one proceed to many other extracts which could with advantage be made from a highly interesting publication, showing clearly that at present, the policy having been placed before us, it is the duty of all connected in any way whatsoever with national health improvement immediately to concentrate upon the unit, great or small, over which they have control and progressively reorganise and improve its working towards the practice of prevention.

